# Clinical Characteristics of Noncancer-Related Upper Back Pain on Initiation of Palliative Care in Patients with Incurable Cancer

**DOI:** 10.1089/pmr.2021.0044

**Published:** 2021-11-17

**Authors:** Hideaki Hasuo, Kiyohiro Sakai

**Affiliations:** ^1^ Department of Psychosomatic Medicine, Kansai Medical University, Osaka, Japan.; ^2^ Department of Psychosomatic Medicine, Faculty of Medicine, Kindai University, Osaka, Japan.

**Keywords:** back pain, cancer, myofascial pain, noncancer pain, palliative care

## Abstract

***Background:*** Cancer patients experience various types of pain unrelated to their malignancy. However, no previous study has reported the prevalence of noncancer-related pain among patients with incurable cancer.

***Objective:*** We aimed to investigate the frequency of noncancer-related upper back pain, the type of noncancer disease, and pain intensity among patients.

***Design:*** This is a multicenter cross-sectional survey.

***Setting/Subjects:*** Subjects were patients with incurable cancer who underwent initiation of palliative care at two university hospitals in Japan.

***Measurements:*** Data for patient characteristics were recorded, and the upper back pain intensity, duration, analgesic use, and opioid drug use with dose were determined. Appropriate statistical tests were also performed.

***Results:*** Among the 103 patients with upper back pain, 20 (19.4%) had cancer-related pain, 28 (27.2%) had both cancer- and noncancer-related pain, and 53 (51.5%) had only noncancer-related pain. Myofascial pain was suspected in the 72 (88.9%) participants with noncancer-related pain. The median pain numerical rating scale score was four in the cancer-related pain group and seven in the other two groups (*p* = 0.005).

***Conclusions:*** A high proportion of outpatients with incurable cancer undergoing palliative care initiation had noncancer-related upper back pain. Severe pain at the initiation of palliative care in patients with incurable cancer may include noncancer-related pain. Trial Registration: UMIN000038371. Registered December 1, 2019.

## Introduction

Patients with cancer experience cancer- and noncancer-related pain, including pain from cancer treatment and pain that is not directly related to cancer. Among cancer survivors, 34.6% have noncancer-related pain, although the types of diseases associated with noncancer-related pain have not been reported to date.^1^ No previous study has reported the rate of noncancer-related pain in patients with incurable cancer. However, 30% of patients with head and neck cancer and 20%–60% of those with breast cancer are reported to experience chronic pain localized at the site of radiation or surgery that was caused by cancer treatment.^2,3^

In addition, 31%–45% of patients with incurable cancer who complained of pain had myofascial pain that was not directly related to cancer.^4,5^ The coexistence of cancer-related pain and myofascial pain at the site of the chief complaint was observed in 64% of patients with advanced cancer.^4^ On the basis of these reports, the incidence of noncancer-related pain in patients with incurable cancer and its comorbidity with cancer-related pain may be high.

This study investigated the frequency of noncancer-related pain, types of noncancer diseases, and the intensity of this pain in patients with incurable cancer who complained of upper back pain at initiation of palliative care.

## Methods

### Study design

This study was a secondary analysis of a multicenter cross-sectional survey that initially investigated the clinical characteristics of alexisomia (which is characterized by difficulties in the awareness and expression of bodily feelings) in patients with incurable cancer at two university hospitals in Japan. The study was approved by the medical ethics committee of our university (reference number: 2019189), and the main study has been published.^6^

### Participants and recruitment

This study included outpatients referred to palliative care services who met the following eligibility criteria: diagnosed with cancer, incurable malignancy, aged 20 years or older, and complaints of upper back pain. The exclusion criteria were any comorbidity related to psychiatric diseases or conditions that made communication difficult (e.g., cognitive impairment or delirium) and refusal to participate. Consecutive patients from each institution between December 2019 and June 2020 were screened, and eligible patients were enrolled in this study. Informed consent was obtained from all the patients.

### Data collection

Patient characteristics were recorded when palliative care was initiated. The information included demographics, site of the primary cancer, Eastern Cooperative Oncology Group performance status, and information about upper back pain. Data related to upper back pain included pain intensity, duration of pain, analgesic use, and opioid drug use with dose. The upper back was defined as the region below the neck and above the costal margin of the eighth thoracic vertebra.

Pain intensity during the previous 24 hours was assessed using an 11-point pain numerical rating scale (PNRS) with scores ranging from 0 (no pain) to 10 (the worst possible pain).^7^ Analgesics included nonsteroidal anti-inflammatory drugs, acetaminophen, analgesic adjuvants, and opioid analgesics. Diagnosis of cancer-related and noncancer-related pain was based on physical examination and imaging (computed tomography, etc.). For cases showing both types of pain at the site of the chief complaint, we considered that both types of pain (cancer-related and noncancer-related pain) were comorbid. All examinations were performed by one of two expert clinicians (H.H. and K.S.), both of whom had >10 years of experience in palliative medicine.

### Statistical analysis

Data are reported as mean with standard deviation (SD), median and interquartile range, or frequency (%), as appropriate. The proportions of participants with only cancer-related pain, both cancer-related and noncancer-related pain, and only noncancer-related pain were calculated. Unpaired one-way analysis of variance was used to compare dependent variables, such as age and pain duration. Pearson's chi-square tests were performed for the statistical analysis of gender, Eastern Cooperative Oncology Group performance status, analgesic use, and opioid drug use. Kruskal–Wallis tests were performed for the PNRS scores and opioid drug doses. Multiple-comparison Bonferroni correction was used for variables that showed significant differences in each test.

## Results

A total of 301 outpatients were referred to palliative care services in the two hospitals, and 116 outpatients met the eligibility criteria for this study. Among these 116 patients, 103 were selected as participants; the other 13 patients were excluded because they showed comorbidities related to psychiatric diseases or conditions that made communication difficult (*n* = 11) or had a life crisis (*n* = 2). The participants' average age was 62.9 years (SD = 12.9 years), and the proportion of males was 49.5% (95% confidence interval: 39.8%–59.2%). The primary cancer sites were gastrointestinal (25/103, 24.3%), liver/pancreas/biliary (22/103, 21.4%), breast (20/103, 19.4%), gynecological (16/103, 15.5%), urological (9/103, 8.7%), head and neck (8/103, 7.8%), and lung (3/103, 2.9%).

Twenty participants (19.4%) were in the cancer-related pain group, 28 (27.2%) were in the cancer- and noncancer-related pain group, 53 (51.5%) were in the noncancer-related pain group, and 2 (1.9%) could not be diagnosed. Cancer-related pain was categorized as pain caused by the primary lesions (13/48, 27.1%), bone metastasis (23/48, 47.9%), lymph node metastasis (9/48, 18.8%), and skin metastasis (3/48, 6.2%). The most commonly identified form of noncancer-related pain was myofascial pain (72/81, 88.9%). There were two cases each of postradiation pain syndrome, postherpetic neuralgia, and facet arthropathy (6/81, 7.4%). The remaining cases included one case each of cervical spondylotic radiculopathy, spinal compression fracture, and fibromyalgia (3/81, 3.7%).

Table 1 gives a comparison of the three groups with different types of pain according to their demographic information, Eastern Cooperative Oncology Group performance status, primary cancer site, and type of noncancer disease with upper back pain. Table 2 gives a comparison of the three groups based on clinical information about upper back pain.

**Table 1.** Comparison of Demographic and Clinical Characteristics in the Three Groups: Cancer-Related Pain, Comorbid Cancer-Related and Noncancer-Related Pain, and Noncancer-Related Pain

	a. Cancer-related pain (*n* = 20), *n* (%)	b. Comorbid pain (*n* = 28), *n* (%)	c. Noncancer-related pain (*n* = 53), *n* (%)	*p*
Age (years), mean (SD)	66.3 (14.1)	62 (11.6)	62.9 (12.9)	0.418
Gender				
Male	11 (55.0)	16 (57.1)	23 (43.4)	0.438
Female	9 (45.0)	12 (42.9)	30 (56.6)	
ECOG PS				
0–2	15 (75.0)	19 (67.9)	40 (75.5)	0.754
3–4	5 (25.0)	9 (32.1)	13 (24.5)	
Primary cancer site				
Lung	1 (5.0)	1 (3.6)	1 (1.9)	
Gastrointestinal	6 (30.0)	8 (28.6)	11 (20.8)	
Liver, pancreas, biliary system	8 (40.0)	7 (25.0)	6 (11.3)	
Breast	1 (5.0)	3 (10.7)	15 (28.3)	
Gynecological	1 (5.0)	5 (17.8)	10 (18.9)	
Urological	2 (10.0)	3 (10.7)	4 (7.5)	
Head and neck	0 (0)	1 (3.6)	6 (11.3)	
Others	1 (5.0)	0 (0)	0 (0)	
Type of disease with upper back pain				
Cancer primary lesion	5 (25.0)	8 (28.6)	0 (0)	
Bone metastasis	10 (50.0)	13 (46.4)	0 (0)	
Lymph node metastasis	5 (25.0)	4 (14.3)	0 (0)	
Skin metastasis	0 (0)	3 (10.7)	0 (0)	
Myofascial pain	0 (0)	25 (89.2)	47 (88.7)	
Postradiation pain syndrome	0 (0)	1 (3.6)	1 (1.9)	
Postherpetic neuralgia	0 (0)	1 (3.6)	1 (1.9)	
Spinal compression fracture	0 (0)	1 (3.6)	0 (0)	
Facet arthropathy	0 (0)	0 (0)	2 (3.7)	
Cervical spondylotic radiculopathy	0 (0)	0 (0)	1 (1.9)	
Fibromyalgia	0 (0)	0 (0)	1 (1.9)	

ECOG PS, Eastern Cooperative Oncology Group performance status; SD, standard deviation.

**Table 2.** Comparison of Clinical Information for Upper Back Pain in the Three Groups: Cancer-Related Pain, Comorbid Cancer-Related and Noncancer-Related Pain, and Noncancer-Related Pain

	a. Cancer-related pain group (*n* = 20), *n* (%)	b. Comorbid cancer-related and noncancer-related pain group (*n* = 28), *n* (%)	c. Noncancer-related pain group (*n* = 53), *n* (%)	*p*	Multiple comparison
PNRS score, median (IQR)	4 (3–6)	7 (7–9.5)	7 (4–8)	0.005	a < b,^**^ a < c^*^
Duration of pain, months, mean (SD)	2 (1.5–3)	4.5 (4–9.5)	4 (2–6)	<0.001	a < b,^***^ a < c^**^
Analgesic drug use	14 (70.0)	28 (100.0)	37 (69.8)	<0.001	a < b,^*^ b > c^**^
Opioid drug use	9 (45.0)	27 (96.4)	21 (39.6)	<0.001	a < b,^***^ b > c^***^
Dose, mg/day,^a^ median (IQR)	30 (20–120)	30 (30–60)	20 (10–20)	<0.001	a > c,^**^ b > c^***^

^a^ Dose of opioids is expressed as the oral dose of morphine (mg/dL). For conversion: parenteral morphine–oral morphine = 1:2, parenteral oxycodone–oral morphine = 1:2, oral oxycodone–oral morphine = 2:3, fentanyl–morphine = 1:100, and oral methadone–oral morphine = 1:8.

^*^ *p* < 0.05; ^**^p < 0.01; ^***^p < 0.001.

PNRS, pain numerical rating scale; IQR, interquartile range.

## Discussion

To our knowledge, this is the first report to investigate the frequency of noncancer-related pain, the types of noncancer diseases, and pain intensity scores in patients with incurable cancer and upper back pain on initiation of palliative care. A high prevalence (78.7%) of noncancer-related pain was noted among patients with incurable cancer and upper back pain when initiating palliative care. The median PNRS score was 7 for the groups with both cancer-related and noncancer-related pain and the group with noncancer-related pain, indicating severe pain.^7^ Noncancer-related pain lasted for as long as four months (chronic pain),^8^ probably because analgesia was not achieved.

For patients with chronic pain but without cancer, the rate of opioid analgesic use was reported to be 41%,^9^ and opioid use for cancer-related pain in this study was not low. In contrast, myofascial pain accounted for 88.9% of all cases of noncancer-related pain in this study. There is no standard treatment for myofascial pain, and local treatments such as trigger point injections are often selected.^4,5,10^ It is important to diagnose each noncancer disease separately, as in this study, not collectively as noncancer-related pain. Additional research will be required to facilitate detailed assessment and treatment of noncancer-related pain.

It is also important to note that comorbid cancer-related pain and noncancer-related pain were present in 27.2% of the patients with incurable cancer and upper back pain. This group showed a high frequency of opioid analgesic use (96.4%) and reported severe pain (median PNRS score of 7 or higher^6^). Thus, the presence of noncancer-related pain should be suspected if opioid analgesics fail to provide relief from severe pain in patients with cancer. Medical examinations in addition to imaging are also important in diagnosing most noncancer pain.

This study had several limitations. First, the sample size was small (a few patients with each cancer type) since we only included patients with incurable cancer referred to palliative care services at two university hospitals; thus, the results cannot be generalized to patients with cancer. Moreover, there was a selection bias because the all participants was patients with cancer referred to palliative care services. Approximately half of the total number of patients at these two facilities were patients with cancer, who were referred to the palliative care service mainly for pain relief and were treated by pain clinicians.

Second, this study used a cross-sectional design and was only conducted at two institutions; data from larger scale studies using more versatile designs are required to validate these findings. Third, we limited our study to upper back pain. We found that lower back noncancer-related pain was difficult to diagnose, but there was no clear evidence for this. Fourth, many noncancer-related pains could not be diagnosed by imaging, and the diagnostic criteria were unclear. Fifth, it was difficult to clearly distinguish between cancer- and noncancer-related pain. Finally, all examinations were performed by one of two expert clinicians, but ideally, two clinicians should have conducted the evaluations, and one other clinician should have confirmed their findings.

## Conclusions

A high proportion of outpatients with incurable cancer who reported upper back pain on initiation of palliative care had noncancer-related pain. Thus, severe pain on initiation of palliative care in patients with incurable cancer may include noncancer-related pain.

## Data Availability

The data that support the findings of this study are openly available at http://doi.org/, reference numbers 9 and 15.

## Abbreviations Used

ECOG PS: Eastern Cooperative Oncology Group performance status
IQR: interquartile range
PNRS: pain numerical rating scale
SD: standard deviation
